# Promises and lies: can observers detect deception in written messages

**DOI:** 10.1007/s10683-016-9488-x

**Published:** 2016-07-08

**Authors:** Jingnan Chen, Daniel Houser

**Affiliations:** 1 Economics Department, Business School, University of Exeter, Exeter, UK; 2 Interdisciplinary Center for Economic Science, George Mason University, Fairfax, USA

**Keywords:** Cheap talk, Deception detection, Trust, Trustworthiness, C91, C72

## Abstract

We design a laboratory experiment to examine predictions of trustworthiness in a novel three-person trust game. We investigate whether and why observers of the game can predict the trustworthiness of hand-written communications. Observers report their perception of the trustworthiness of messages, and make predictions about the senders’ behavior. Using observers’ decisions, we are able to classify messages as “promises” or “empty talk.” Drawing from substantial previous research, we hypothesize that certain factors influence whether a sender is likely to honor a message and/or whether an observer perceives the message as likely to behonored: the mention of money; the use of encompassing words; and message length. We find that observers have more trust in longer messages and “promises”; promises that mention money are significantly more likely to be broken; and observers trust equally in promises that do and do not mention money. Overall, observers perform slightly better than chance at predicting whether a message will be honored. We attribute this result to observers’ ability to distinguish promises from empty talk, and to trust promises more than empty talk. However, within each of these two categories, observers are unable to discern between messages that senders will honor from those that they will not.

**Electronic supplementary material:**

The online version of this article (doi:10.1007/s10683-016-9488-x) contains supplementary material, which is available to authorized users.

## Introduction

Economic and social relationships often involve deception (e.g., Gneezy 2005; Mazar and Ariely 2006). Such relationships are generally governed by informal contracts that require trust (Berg et al. 1995). While trust is essential to an economy, the knowledge of who and when to trust, i.e. deception or trustworthiness detection, is equally critical (see, e.g., Belot et al. 2012). In particular, trust is critically important in cases where an exchange can lead to gains, but there are also incentives for one side to defect and appropriate the surplus. In these situations, people may send informal “promises” of future behavior. These messages must be interpreted to gauge the extent to which they can be trusted.

Substantial research has focused on deception in economics (see, for example, Hao and Houser 2013; Erat and Gneezy 2011; Rosaz and Villeval 2011; Kartik 2009; Sutter 2009; Dreber and Johannesson 2008; Charness and Dufwenberg 2006; Ellingsen and Johannesson 2004). Recent research has devoted increasing attention to the question of whether it is possible to detect deception or trustworthiness^1^;(see, e.g., Belot et al. 2012; Darai and Grätz 2010; Konrad et al. 2014). While there have been important advances, previous studies have focused largely on face-to-face communication. To our knowledge, no studies in economics have focused on detecting deception in informal written communication.^2^ This is unfortunate, as informal written communication (e.g., via email, texting, tweeting, or facebooking) plays an increasingly important role in social and economic exchange outcomes. One example is Internet dating,^3^ where interactions often begin with initial informal written message exchanges. The purpose of these exchanges is to build a foundation of mutual trust upon which a real (as compared to virtual) relationship can develop^4^ (Lawson and Leck 2006). Evidently, during this process of written exchanges, each party must make decisions regarding the trustworthiness of the other. Consequently, it is an increasingly important skill for users to be able to write trustworthy-sounding messages, as well as to be able to detect insincere messages.

There is a wide body of literature studying informal communication within the context of “cheap talk”^5^ (see, e.g., Farrell and Rabin 1996; Crawford 1998). Nonetheless, the literature has focused heavily on how cheap talk affects senders,^6^ and very little on how it affects receivers (see, for example, Farrell and Rabin 1996; Croson et al. 2003; Charness and Dufwenberg 2006). If cheap talk messages work by changing receivers’ beliefs about senders’ actions (as suggested by Charness and Dufwenberg 2006), then many important questions remain open. Such questions include: (i) the precise nature of messages to which people are most likely to respond positively; and (ii) the extent to which people are able to distinguish truthful messages from deceptive ones (and correctly update their beliefs). This paper takes a step toward answering these questions. In particular, we investigate whether there are cues that can predict whether a written communication is dishonest, and if so, whether the person reading the message can detect and correctly use those cues.

Our study introduces a novel variant of the trust game (building on the hidden action game of Charness and Dufwenberg 2006). Our game captures an environment with misaligned incentives and opportunities to defect, but also includes potential gains from cooperation. In this context, we offer participants the opportunity to communicate with one another using hand-written messages. We use this design to accomplish three research goals: (i) to determine the characteristics of cheap talk messages that promote receivers’ trust; (ii) to discover objectively quantifiable cues for differentiating promises writers are likely keep from those they are likely to break; and (iii) to assess whether message receivers recognize and respond correctly to those cues.

We find that receivers are significantly more likely to consider longer messages to be promises, as compared to shorter messages. In this sense, there is a payoff to a message sender’s effort. Second, we find that promises mentioning money are significantly more likely to be broken. Yet receivers fail to respond to this cue. Instead, they place more trust in longer promises, despite the fact that senders are just as likely to break such promises as they are to break shorter promises. Finally, people perform, on average, slightly better than random guessing at judging whether a sender will honor a message. The reason is that readers are able to distinguish promises from empty talk, and they correctly place more trust in promises. However, within kept and broken promises, readers cannot reliably determine which promises a sender will or will not honor.

These findings help to explain features of our natural environment. For example, advertisements often provide extensive details regarding the benefits of offered products. Presumably, the reason is that companies have learned that longer promises are more likely to be believed.

This paper proceeds as follows. In Sect. 2, we discuss related literature. Section 3 explains the context from which we obtain the message data, as well as the experimental design. In Sect. 4, we report our analysis and results. Section 5 summarizes and concludes.

## Related literature

Research on deception detection has appeared in both psychology and economics. Key findings from economics indicate that people notice and respond to some cues (for example, gender and presence of a handshake), but not others (e.g., participants’ past behavior) (see, e.g., Belot et al. 2012; Darai and Grätz 2010; Wang et al. 2010; Belot and van de Ven 2016). These results, however, are based only on face-to-face communication. The psychology literature studies the same question, but within the context of qualitative cues, such as facial movements or expressions (e.g., Ekman 2009b). The main finding from this literature is that people do not know what to look for to identify cheating, and consequently perform poorly—not much better than chance—at detecting deception.^7^ In addition, DePaulo et al. (2003) pointed out the participants in psychology studies are typically not incentivized, making it difficult to know whether poor deception detection results from poor “acting” by the deceivers.

The paper closest to ours is Belot et al. (2012). The authors report that subjects in an economic experiment were able to use some objective cues (while ignoring others) to improve their ability to detect deception and trustworthiness. The authors made a novel use of data from a high-stakes prisoner’s dilemma game show. Subjects watched clips and rated the likelihood that players would cooperate pre- and post-communication. The authors discovered that subjects were able to use some^8^ objective features of the game’s players (such as gender and past behaviors) to make pre-communication predictions. Although subjects did not seem to improve their overall predictions after observing communication between the players, they did respond positively to the “elicited promise”^9^ communication group. The authors concluded that previous research might have underestimated people’s ability to discern trustworthiness in face-to-face interactions. Another related study is Utikal (2013), where the author looks into the differential effect of truthful and fake apology on forgiveness with typed messages. The author finds that people seem to be able to distinguish truthful and fake apologies, and are more likely to forgive after truthful apologies.

In sum, most research to date has emphasized people’s ability to detect deception or trustworthiness in face-to-face encounters. Face-to-face interaction is a very rich and relevant environment for assessing people’s ability to detect deception; however, the environment may be too complex to enable one to draw inferences as to the reasons for people’s performance. Many factors are at play, including facial expressions, body movements, hand gestures and language. Many of these factors are quite hard to measure. Consequently, it can be difficult in these studies to pinpoint the information people acquire and use.^10^ For example, in Belot et al. (2012), the authors show that subjects are able correctly to predict females as relatively more trustworthy than males. There are many possible explanations for this. It may be that: (i) females are more sensitive to guilt, and thus less likely to lie (and more trustworthy in general) (e.g., Dreber and Johannesson 2008; Erat and Gneezy 2011); or (ii) females are less capable of concealing their emotions in facial expression (e.g., Papini et al. 1990), and thus are more likely to be considered trustworthy by observers.

Further, prior research has not systematically investigated the ability to predict trustworthiness through other forms of communication^11^ (e.g., online written communication such as that used in dating websites), despite their ubiquity and importance. This paper contributes to the literature by using a controlled laboratory experiment to investigate cues that predict deception (untrustworthiness), and to offer explanations as to why people detect or fail to detect untrustworthiness. Relatedly, our analysis offers new insights into how to convey trustworthiness.

## The game, messages and evaluations

### The Mistress Game^12^

**Fig. 1:**
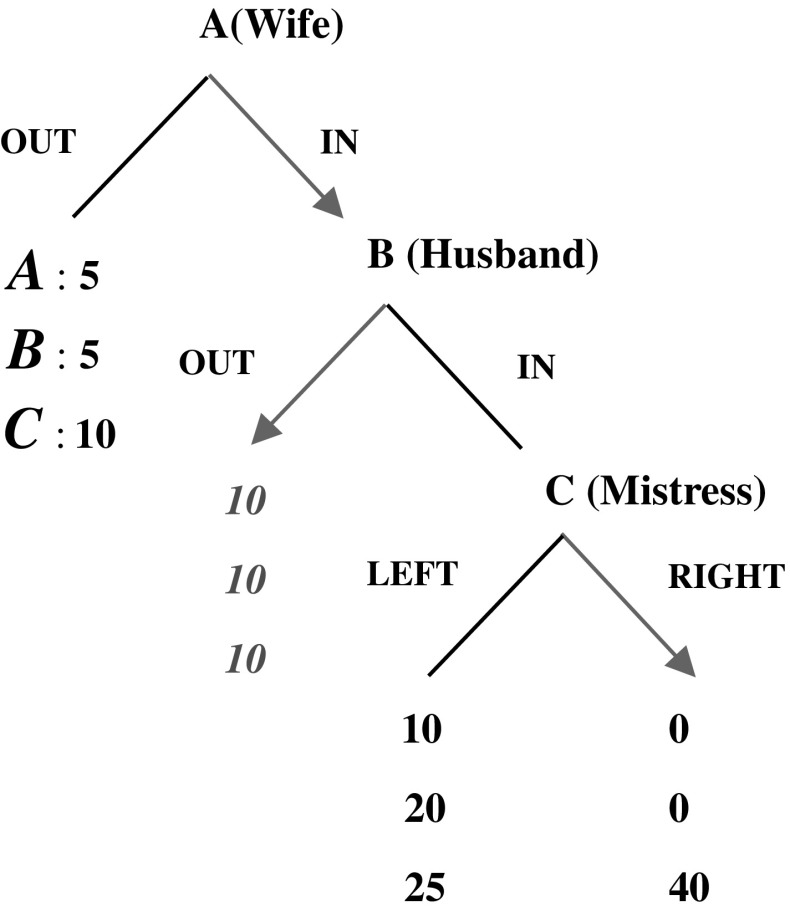
The Mistress Game

We devised a novel three-person game^13^ to generate written messages. Third party observers in a subsequent experiment then evaluated these messages. They were asked to assess the nature of the message (e.g., a promise or empty talk) and predict the behaviors of the message senders, as detailed in Sect. 3.3.^14^ The extensive form of the Mistress Game is shown above in Fig. 1. Payoffs are in dollars.

The Mistress Game builds on the hidden action trust game (Charness and Dufwenberg 2006), but chance (the die roll) is replaced with a strategic third player C in our game. Our payoff structure offers incentives that suggest the following interpretation.

A and B consider whether to form a partnership; if no partnership occurs, then both parties receive the outside option payoff of $5. At this point, C is not relevant and receives $10 as the outside option. If a partnership is formed, a trust relationship emerges, and the payoffs to this relationship depend on the B’s decision. B is faced with a dilemma—either to stay with the current trust relationship (corresponding to B’s *Out* option) or form an additional trust relationship with a third person (at this decision point, C is now relevant) and enjoy a potentially higher payoff (corresponds to B’s *In* option). Note that A is NO better off (maybe even worse off) by B’s choosing *In*; therefore, A would always prefer B to choose *Out* and maintain an exclusive partnership. If B chooses to stay with A [corresponding to the strategy profile (*In, Out, Left/Right*)], both A and B are better off (with the payoff of $10 for each), and C (who has no move) again earns the outside option of $10. The strategy profile (*In, Out, Left/Right*) corresponds to the situation where an exclusive partnership contract is enforceable. However, such a contract may not be enforceable. Indeed, B’s choice may not be observable to A, depending on C’s decision. Our game captures this as discussed below.

If B chooses to form a new trust relationship with C (corresponding to B’s *In* option), C can either be cooperative and reciprocal by choosing *Left*, or defect by choosing *Right*. Note that if C chooses *Left*, B’s behavior is unknown to A (B’s original partner). However, if C chooses *Right*, not only does B receive nothing from the newly-initiated trust (C takes all), A is also impacted and receives nothing. In this case, A knows B’s choice. Note that A may foresee such outcomes and choose not to enter a trust partnership with B. The players’ choices, *Out, In* and *Right*, describe those possibilities. It is easy to verify that the sub-game perfect equilibrium of this game for selfish and risk-neutral players is (*In, Out, Right*), which is also inefficient.

### The messages

In addition to the regular no-communication game play, we also introduce one-sided pre-game communication to the environment: the players have an opportunity to send a handwritten note to their counterparts. In particular, for the purpose of this paper, we focus on the messages from C to B under two different environments: single message and double message.^15^

#### Single message environment

**Fig. 2:**
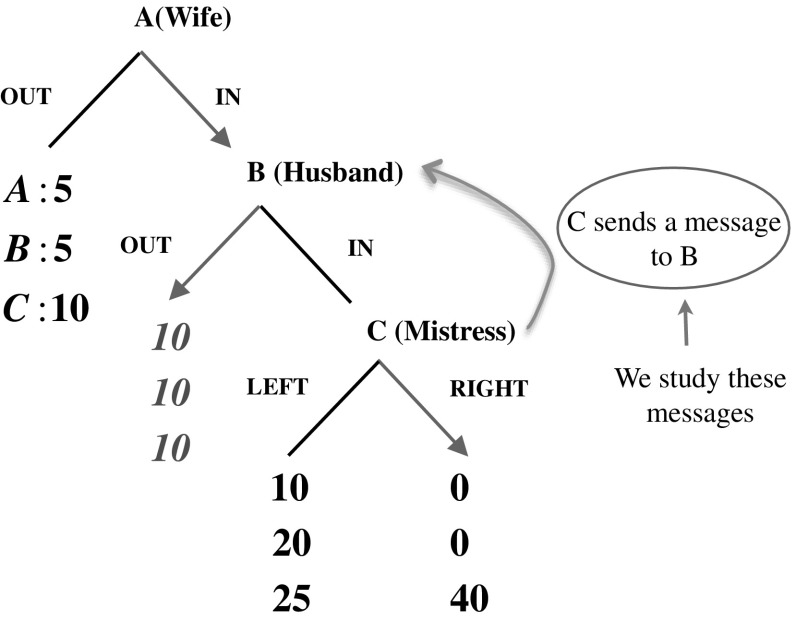
The single message communication phase

Before the subjects play the Mistress Game, C has the option of writing a message to B. The experimenter then collects the messages and passes them as shown in Fig. 2. That concludes the communication phase, and the subjects start to play the game.^16^

#### Double message environment

**Fig. 3:**
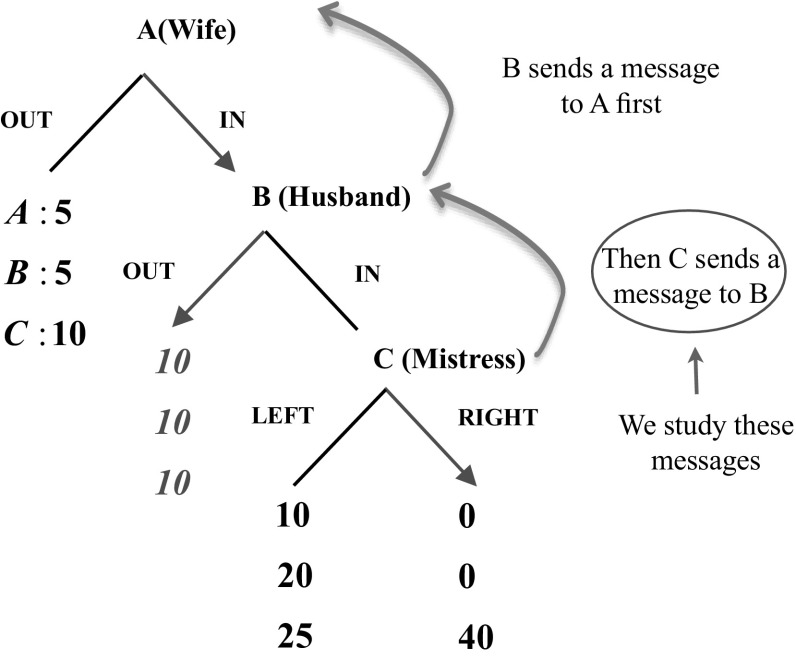
The double message communication phase

As shown in Fig. 3, the double message environment is similar to the single message environment, except that the opportunity for C to send a message to B comes as a surprise.

It is common knowledge from the beginning of the experiment that B has an opportunity to send a hand-written message^17^ to A. After the messages are transmitted, the experimenter announces a surprise message opportunity: C can also send a message to B. The experimenter waits for the Cs to write their messages and then passes the messages on to their paired Bs. Upon completion of the message transmission, subjects start to play the game.

In both the single and double message environments, C is better off when the B chooses *In*; therefore, it is natural to assume that the C would use the messages as a means to persuade B to choose *In*. However, the two environments also depart significantly from each other.

Specifically, in the double message environment, where everyone knows that B has already sent a message to A, it is reasonable to presume that B might have conveyed his intention to stay with A and might choose *Out*. Therefore, it is very likely that C needs to do a better job in convincing B to choose himself/herself instead by choosing *In*. Indeed, we find some evidence suggesting that C worked harder in crafting their messages, as messages are significantly longer in the double message environment.

### The experiment

#### Design and procedure

The evaluation sessions were conducted in the experimental laboratory of Interdisciplinary Center for Economic Science at George Mason University.^18^ We recruited 93 evaluators from the general student population (22 evaluators to evaluate messages from single message environment and 71 to evaluate messages from double message environment). None of the evaluators had previously participated in the Mistress Game experiment. Average earnings were $18 (including the $5 show-up bonus); sessions lasted about 1 h.

Before reviewing any messages, evaluators were acquainted with the Mistress Game and provided with a transcript of the Mistress Game instructions for either the single message environment or the double message environment. A quiz was administered to ensure that all the evaluators understood their tasks, as well as the context in which the messages would be written.

There were, in total, 20 and 60 messages collected from the Mistress Game single and double message sessions respectively^19^. All of the messages were scanned into PDF files and displayed on the computer screen in random order for the evaluators to look through. Each evaluator worked on all messages independently inside their own visually-separated cubicles. They were not provided with any information regarding the decisions of the message-senders or their partners. Nor were the evaluators given any information regarding the purpose of the study, or the hypotheses of interest. Evaluators were instructed to first classify each message as either “Promise or Intent” or “Empty Talk,” and then to make conjectures as to what the message senders actually did.

To clarify the meanings of “Promise or Intent” and “Empty Talk,” we provided the following statement in the instructions^20^:

… A message should be categorized as a statement of intent or promise if at least one of the following conditions is probably satisfied:

1. the writer, subject C, indicates in the message he/she would do something favorable to subject B or refrain from doing something that harms subject B; or.
2. the message gives subject B reasons to believe or expect that subject C would do something favorable to subject B or refrain from doing something that harms subject B.

A message should be coded as empty talk if none of the above conditions are satisfied…

We followed the XH classification game^21^ (Houser and Xiao 2010) to incentivize the first evaluation task: two messages were randomly chosen for payment, and the evaluators were paid based on whether their classifications coincided with the median choice of the evaluation group. This was essential, as the average opinion of a large number of evaluators who are also strangers to the message writer is a reasonable way to infer not only how the message was likely interpreted, but also the way in which the message writer expected the message to be interpreted. This is especially true when the evaluators are from the same pool as the message writers and receivers.

For the second task, another two messages were randomly chosen for payment, and evaluators were paid based on whether their guesses matched the actual behavior of the message senders. Upon completion of the evaluation tasks, the evaluators were given a survey with questions that evaluated things like how they made their classification or guess decisions. The experimental instructions are available as an appendix to this paper.

#### Cues and their effects

One advantage of written messages compared to face-to-face communications is that they have fewer cues that one can make use of and quantify. In view of the literature, we developed several conjectures regarding cues in written messages that may impact the perceived trustworthiness of the messages:

### Mention of money

The mention of money may impact how evaluators assess the trustworthiness of a message in a positive way. The reason is that the mention of money contains information that is relevant to game play, and thus gives credibility to the message. This may make the sender seem more trustworthy. Consequently, the message is more likely to be evaluated as a promise (see, e.g., Rubin and Liddy 2006).

### Use of encompassing words

The use of encompassing words can foster a common social identity among message senders and receivers (Hall 1995). This sort of “in-group” effect can impact the sense that a message is a promise, as well as the belief that a promise will be kept. Indeed, being part of an in-group can also impact reciprocity decisions. A rapidly growing literature supports these observations. For example, Kimbrough et al. (2006) found that it is more common to mention “we” or “us” during chat with in-group rather than out-group members, and that the mention of these encompassing words is positively correlated with cooperation and the willingness to make and keep promises to do personal favors. Schniter et al. (2012) concluded from their experiments that one of the steps for effectively restoring damaged trust with a partner is to convey “a shared welfare or other-regarding perspective.”

### Message length

According to the heuristic model, the structural or surface attributes of the message may be processed in a heuristic manner (Chaiken 1980). If strong and compelling messages are often associated with longer and more detailed arguments, people may learn a rule suggesting that length implies strength. Application of this heuristic would then suggest longer messages being more persuasive than short ones. Indeed, there are some evidence in support of this theory (see, e.g., Petty and Cacioppo 1984). Therefore, longer messages are more likely to be perceived as promises and trusted by the receivers.

### Gender of the message writer

We do not expect gender of the message writers to impact the message evaluation. The evidence for gender differences in perceived trustworthiness/honesty is quite divided (for a review, see, Buchan et al. 2008). In some studies, males are viewed as more trustworthy than females (Jeanquart-Barone and Sekaran 1994); in other studies, females are believed to be more trustworthy/honest (Wright and Sharp 1979; Swamy et al. 2001); some studies fail to find any significant perceived trustworthiness difference between males and females (Frank and Schulze 2000).

## Results

### Receivers behaviors in mistress game: the power of words

To demonstrate the significant impact of communication on the receivers (Role B), we present below the decisions made by B in the Baseline (no messages were sent), Single and Double treatments.

**Fig. 4:**
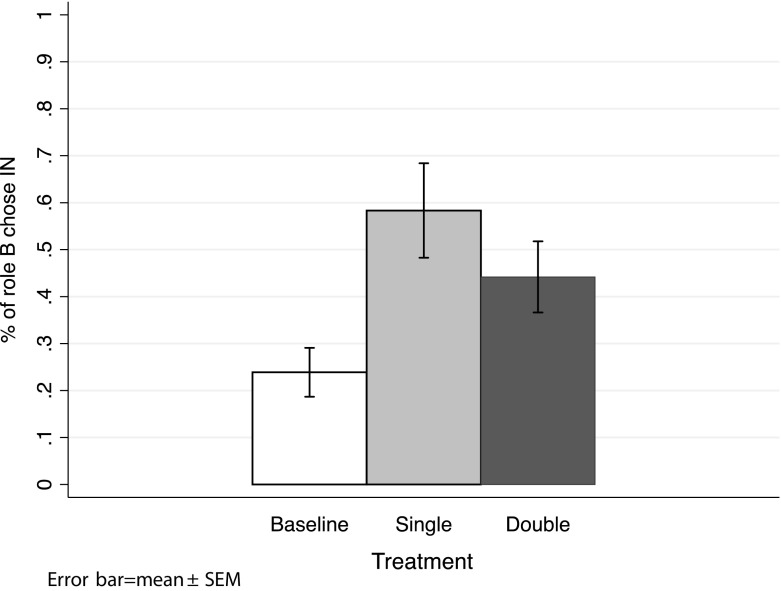
Role B decisions

As shown in Fig. 4, only 24 % of B chose *In* in the Baseline treatment. By contrast, in the Single treatment, 68 % chose *In*, and in the Double treatment, 52 % chose *In*. These differences (Single vs. Baseline and Double vs. Baseline) are statistically significant at the 1 % level. Having established that communication significantly impacts decisions in our game, we now address our central question: can observers detect deception?^22^

### Evaluation: Data and descriptive statistics

We obtained 80 messages in total from the communication phase of the Mistress Game: 20 messages from Single, and 60 from Double, all of which were classified by our evaluators. Among the 20 messages from Single, 80 % were categorized as promises or statements of intent^23^; 77 % of the 60 messages from Double were classified as including a promise or intent^24^,^25^ (See Table 1).

**Table 1:** Message evaluation results

	Single msg	Double msg
Promises/statements of intent	16 (80 %)	46 (77 %)
Empty talk	4 (20 %)	14 (23 %)
All messages	20	60

The messages from both environments are statistically identical in terms of mentions of money, mentions of we/us, and the gender of the message sender. However, they differ in terms of message length. As shown in Table 2 above, around a quarter of the messages include money mentions, and less than one third involve the use of “we,” “us” or “let’s.” Messages from Double are significantly longer than those from Single. This may stem from the fact that in the double message environment, C understands that B communicated with A, and thus it may be more difficult to convince B to select *In*. Consequently, Cs exert more effort and write longer messages.

**Table 2:** Comparison of the messages from single and double environment

Environment	Observations		Mean		Z stat
	Single	Double	Single	Double	
Mention of money^a^	20	60	0.20 (0.09)	0.32 (0.06)	0.99
Mention of “we/us”^b^	20	60	0.20 (0.09)	0.33 (0.06)	1.12
Word count^c^	20	60	7.85 (1.43)	14.15 (1.61)	1.87*
Male sender^d^	20	59^e^	0.70	0.68	0.18

Standard errors are reported in the parentheses. The Z statistic derives from two-sided Mann–Whitney tests

* p < 0.10, two tailed tests

^a^Mention of money is a binary variable; it is coded as 1 if there is any money/payoff related discussion in the message (payoff for the game, benefit from the game, and so on) and 0 otherwise

^b^Mention of we/us is also a binary variable: = 1 if the message sent used “we,” “us” or the abbreviated form, e.g., “let’s,” and 0 otherwise

^c^Word Count is the number of words in the messages

^d^Male is a binary variable: it is set to unity if the gender of the message sender is male, and to zero otherwise

^e^One subject indicated that s/he is bi-gender

### Perceived cues for trustworthiness from the receivers

We begin this section by investigating the type of messages more likely to be regarded as promises (Sect. 4.3.1). We proceed to examine the cues that influence the perceived trustworthiness of a message, as well as the cues that predict actual trustworthy behaviors (Sect. 4.3.2). Interestingly, we discover that whether a message is coded as a promise is a significant predictor not only of perceived trustworthiness, but also of actual trustworthy behavior. Finally, in order to better understand this phenomenon, we provide an analysis narrowly focused on promises (Sect. 4.3.3).

#### What makes a promise?

In this section, we investigate objective features that receivers perceive as indicative of more trustworthy messages. In particular, we attempt to discover whether any of the objective features of the messages discussed above are significantly (positively or negatively) correlated with whether the message was classified as a promise, and, if so, the extent to which that promise is trusted.

We begin by pooling the message classification data from the first task,^26^ and then analyzing those data using a Tobit regression model. In this analysis each message is treated as an independent observation, and the dependent variable is the frequency with which each message is categorized by the evaluators as a promise (thus the dependent variable is censored from below at 0 and from above at 1). This frequency is regressed on whether money is mentioned in the message, whether there is a mention of “we” or “us” in the message, the number of words in the message, and the gender of the message writer. We report the results in Table 3.

**Table 3:** Tobit regression of message classification on perceived cues

Dependent variable: frequency considered as promise	(1)	(2)
Mention of money	.03 (.06)	.03 (.06)
Mention of we/us	.04 (.06)	.07 (.06)
Word count	.008*** (.003)	.008*** (.003)
Male		−.09 (.06)
No. of observation	80	79^a^

Robust Standard errors are reported in parentheses

*, **, *** Correspond to 1, 5 and 10 % significance level, respectively

^a^We lose one observation by adding $male$ as a regressor, because one of the message sender indicates that he/she is bi-gender

Table 3 suggests that, when coding the messages as either Promise or Empty Talk, our receivers seem to rely primarily on the length of the messages: all else equal, longer messages are significantly more likely to be considered promises.^27^

#### What predicts perceived trustworthiness?

Next, we consider messages coded as promises by the majority of the evaluators. Our goal is twofold: (1) to understand the cues that are used by the evaluators in guessing whether a promise is likely to be trusted; and (2) to compare the perceived cues with the actual cues that predict senders’ behavior.

We use a Tobit regression to analyze the pooled guessing data from the second task.^28^ The unit of observation is the message, and the dependent variable is the frequency with which message *i* is trusted by the evaluators (censored at 0 and 1). The regressors include those reported in Table 3, as well as two additional variables. One is *Promise.* This is a dummy variable taking value 1 if message *i* is coded as a promise by a majority of the evaluators, and is zero otherwise. The second new regressor, *Promise Broken* is the product of *Promise* and *Broken*. The latter is a dummy variable that takes value 1 if the sender of the message chose *Right*; and is zero otherwise.

We describe the regression results in Table 4. From regression (1) and (2), one discovers that receivers use length of the message: longer messages are significantly more likely to be trusted, everything else equal.^29^ From (3), we find that promises are significantly more likely to be believed. On average, a promise is 41 % more likely to be trusted compared to empty talk, ceteris paribus. Finally, as shown in (4), although receivers put significantly more trust in promises, that trust is often misplaced, as the readers cannot distinguish promises that will be kept from those that will be broken.

**Table 4:** Tobit regression of perceived cues for trustworthiness using all messages

Dependent variable: frequency of trust for messages	(1)	(2)	(3)	(4)
Mention of money	−.005 (.05)	−.005 (.05)	−.03 (.03)	−.02 (.03)
Mention of we/us	.04 (.04)	.05 (.04)	.002 (.04)	.007 (.04)
Word count	.008*** (.002)	.008*** (.002)	.003** (.001)	.003** (.001)
Male		−.03 (.05)	.04 (.03)	.04 (.03)
Promise			.41*** (.04)	.41*** (.04)
Promise broken				−.02 (.03)
No. of observation	80	79	79	79

Robust standard errors are reported in parentheses

*, **, *** Correspond to 1, 5 and 10 % significance level, respectively

Now we turn to the cues that predict senders’ actual decisions. We conducted bivariate probit regressions using decision data from actual message senders. The unit of observation is again the message. The dependent variable is binary, taking value 1 if the sender of message *i* chose *Left* (the cooperative option) and zero otherwise. As detailed in Table 5 below, we find that the only cue that predicts senders’ cooperative decisions across all messages is whether the message is coded as a promise. The senders who made a promise are significantly more likely to choose the cooperative option (*Left*) than the empty talk senders. That is, senders who made a promise choose to cooperate substantially more frequently than those senders who did not send a promise.

**Table 5:** Actual cues predicting senders’ behavior using all messages

Dependent variable: senders’ actual decision	(1)	(2)	(3)
Mention of money	−.21 (.16)	−.20 (.16)	−.22 (.17)
Mention of we/us	−.14 (.16)	−.15 (.16)	−.21 (.18)
Word count	.004 (.006)	.003 (.006)	−.003 (.006)
Male		.10 (.12)	.19 (.12)
Promise			.41*** (.13)
No. of observation	80	79	79

Marginal effects are reported, robust standard errors in parentheses

*, **, *** Correspond to 1, 5 and 10 % significance level, respectively

From the evaluators’ perspectives, longer messages and promises are more likely to be trusted (Table 4). Although longer messages do not correspond to more trustworthy behavior, promises do predict that the message sender will be more trustworthy (Table 5). In the next section, we analyze messages coded as a promise in greater detail.

#### Perceived cues for trust: promises

Table 6 describes the relationship between characteristics of promises^30^ and evaluators’ guesses. The dependent variable is the frequency with which *promise message* *i* is trusted by the evaluators. We find that evaluators are significantly more likely to trust the promise when it is longer. For example, a promise with 10 additional words is 3 percentage points more likely to be trusted, all else equal.

**Table 6:** Tobit regression of perceived cues and trust using promises

Dependent variable: frequency of trust for promises	(1)	(2)
Mention of money	−.02 (.03)	−.01 (.01)
Mention of we/us	−.01 (.03)	−.01 (.04)
Word count	.003*** (.001)	.003*** (.001)
Male		0.02 (.03)
Number of observations	62	61

Robust Standard errors are reported in parentheses

*, **, *** Correspond to 1, 5 and 10 % significance level, respectively

*Actual cues for trustworthiness*: *promises:* We now turn to an analysis of promise senders’ actual decisions. As shown in Table 7, broken promises are more likely to mention money, use more encompassing words, and also include more words.

**Table 7:** Actual cues for promises

	Promise		Z Stat
	Kept	Broken	
Mention money	.19 (.07)	.58 (.10)	3.08***
Mention “we/us”	.22 (.07)	.58 (.10)	2.83***
Word count	12.64 (1.57)	18.27 (2.79)	1.73*
Male	.66 (.08)	.65 (.10)	0.03
Observations	36	26	

The Z statistic derives from two-sided Mann–Whitney tests of the null hypothesis that means in Kept and Broken are identical

*, **, *** p < 0.10, 0.05 and 0.01, respectively, two-tailed tests

We then control for possible partial correlations among cues. And the results are reported in Table 8 below. Regression (1) uses a Probit analysis with dependent variable taking value 1 if the sender of message *i* chose *Left* (the cooperative option) and zero otherwise. Regression (2) reports the results of a Tobit regression with dependent variable equal to the frequency with which *promise message* *i* is trusted by the evaluators. In both cases the independent variables are those described in Table 3.

**Table 8:** Actual cues versus perceived cues for promises

Dependent variable: cooperative decision	Actual realization (1)	Evaluators’ prediction (2)
Mention of money	−.25*** (.01)	−.01 (.01)
Mention of we/us	−.22 (.17)	−.01 (.04)
Word count	−.004 (.002)	.003*** (.001)
Male	.13 (.08)	0.02 (.03)
No. of observation	61	61

Robust standard errors are in parentheses

* and *** Correspond to 10 and 1 % significance levels, respectively. Column 1: bivariate probit estimates, marginal effects. Column 2: Tobit estimates

The results from regression (1) make clear that mention of money is the single best predictor of senders’ defections. In particular, Cs are 25 % more likely to defect when they mention money in their messages. Our evaluators, however, identified only word count as a positive indicator of senders’ trustworthiness. Message length, on the hand, does not seem to suggest greater trustworthiness.

The reason that the mention of money is the single best predictor of senders’ decisions to defect may be that the mention of money may “monetize” the exchange. Such an effect is suggested by a sizable “crowding out” literature (see for example, Ariely and Bracha 2009; Lacetera and Macis 2010; Mellstrom and Johannesson 2008; Gneezy and Rustichini 2000a, b; Fehr and Falk 2002; Li et al. 2009; Houser et al. 2008). This literature emphasizes the idea that monetizing choices may crowd out extrinsic incentives, shift decision-makers’ perception of the environment into a “business” frame, and focus their attention on self-interested decision-making. Additionally, Vohs et al. (2006) suggested that “money brings about a self-sufficient orientation”: when subjects are primed with money, they tend to be less helpful towards others.

### Cues and predictions

Table 9 below reports the results of evaluators’ guesses regarding whether the message would be believed to lead to a cooperative action, and also whether the subsequent action was actually cooperative. We divide the messages into two groups: Promises and Empty talk. We find that among the Promises, 71 % of evaluators believed that message senders would keep their promise (choose *Left*). This belief is statistically different from the actual rate—overall 58 % of promises were kept. We find further support for this result when we look into promises that include mentions of money, encompassing terms, or are longer than median length. In all these cases, evaluators were over-optimistic that the promise would be kept: differences between evaluators’ beliefs and actual behavior are statistically significant in these cases. In contrast, for messages identified as empty talk, only 28 % of the evaluators believed that the message sender would cooperate. This is statistically indistinguishable from the one-third of senders who did actually choose *Left*. Moreover, beliefs are statistically correct in all of three sub-categories of the empty talk messages.^31^

**Table 9:** Predictions by receivers: summary statistics

Message type	Obs	Average rate of trust^a^	Actual rate of cooperation^b^	T-stat^c^	Rate of accuracy^d^
Promises	62	.71 (.01)	.58 (.06)	1.97**	.53 (.03)
Money mention = 1	22	.71 (.02)	.32 (.10)	3.81***	.43 (.05)
Us mention = 1	23	.71 (.02)	.35 (.10)	3.55***	.46 (.05)
Word count = Long	38	.72 (.02)	.47 (.08)	2.98***	.52 (.04)
Empty talk	18	.28 (.04)	.33 (.11)	0.41	.62 (.06)*
Money = 0	17	.28 (.04)	.29 (.11)	0.05	.64 (.05)**
Us = 0	17	.27 (.04)	.29 (.11)	0.19	.63 (.06)*
Word count = Short	16	.27 (.04)	.25 (.11)	0.14	.64 (.06)**
	80	.61 (.02)	.53 (.06)	1.44	.56 (.03)*

Standard errors are in the parenthesis

*, **, *** p < 0.10, 0.05 and 0.01, respectively

^a^The average prediction of the percentage of the population that believes the message is honored

^b^Actual rate of cooperation is defined as the percentage of messages that are followed by a cooperative move from the message sender

^c^The statistics reflect the two-sided *t* test for the null hypothesis that the Average Prediction and Actual Rate of Cooperation are equal

^d^The asterisk indicates significance of two-tailed tests under the null hypothesis that the rate of success is $A_{random} = .5056$

Regarding the accuracy rate measured by the average percentage of correct guesses for all evaluators, 57 % were able to make correct predictions based on the messages (i.e., their guesses match the actual senders’ choices). However, when considering messages categorized as promises, 53 % of evaluators were able to make the correct predictions, while 62 % predicted the sender’s decisions correctly for empty talk messages. It is clear where mistakes were made: evaluators placed higher trust in promises that mentioned money than in those that did not, while at the same time those messages were least likely to be honored. In contrast, empty talk messages that neither mentioned money nor used encompassing words were trusted less by evaluators (as were shorter messages). Consequently, the evaluators achieved higher rates of accuracy in those cases.

We now turn to an analysis of the accuracy of evaluators’ guesses. As an accuracy benchmark we use the average accuracy expected under random guessing. Any given message will be trusted by receivers with probability 0.61 (as measured by the average rate of trust, see Table 9 last row third column). Further, receivers are correct with probability 0.53 (as measured by the average actual rate of cooperation for all messages, Table 9 last row forth column). Therefore, random guessing results in accuracy rate 0.61 × 0.53 + (1 − .61) × (1 − .53) = .51. Formally, the accuracy of random guessing for any message *i* is calculated as follows:

$$ A_{random} = P\left( {trust} \right) \times P\left( {Left} \right) + \left[ {1 - P\left( {trust} \right)} \right] \times \left[ {1 - P\left( {Left} \right)} \right] $$

where *P*(*trust*) is the percentage of the population that trust the message *i* and *P*(*Left*) is the average actual rate of cooperation for any message *i*. *P*(*Left*) also represents the average probability that the evaluator’s trust is correct.

When we compare the all-message accuracy rate against $A_{random} = .51$, we find that on average our evaluators are slightly better than random guesses at a 10 % significance level. However, for promises, our evaluators are not any better than random guesses, especially for promises that mention money; for empty talk, however, evaluators are significantly better than random guesses, with the average accuracy rate of 63 % (12 % higher than the random guess benchmark). This suggests that readers are able to distinguish between promises and empty talk and treat those two types of messages differently and correctly, by putting greater trust in promises than empty talk. However, readers cannot differentiate the kept and broken messages within each type of messages (as detailed in Tables 4, 8).

## Discussion

This paper focuses on the importance of understanding cues for deception (or honesty) in natural language written messages. It is well established that people respond to cheap talk communication. We conducted a laboratory experiment in which people could offer written promises of cooperative actions. The messages were evaluated by independent observers. We contribute to the literature by using these evaluations, as well as the behaviors we observed in the game, to shed light on: (i) whether there exist objective cues that correlate with a message sender’s likelihood of breaking a promise; (ii) the nature of any such cues; and (iii) whether message receivers recognize and respond to cues correctly.

We found systematic evidence that: (i) people place greater trust in longer messages and messages they consider to be “promises”; (ii) promises that mention money are significantly more likely to be broken; and (iii) people do not respond to the mention of money correctly, in that they are more likely to trust these messages. Overall, we find that people perform slightly better than random chance in detecting deception. The main explanation is that our evaluators are able to differentiate between promises and empty talk correctly, and trust promises more than empty talk. However, within the promise and empty talk groups, readers are not able to distinguish messages that will be honored from those that will not.

It is worthwhile noting that we used hand-written messages in the original game experiment, and it seems important for our evaluators to see what our participants saw while making decisions in the game to minimize experimenter demand effect. With respect to the original game sessions, we thought that hand-written messages might seem more “real” and meaningful than typed messages (the same reason that Xiao and Houser 2005, 2009; Houser and Xiao 2010) used hand-written messages in their analyses). Further, it is not obvious that typed messages are less gender-identifiable than written messages. Our own experience is that men and women tend to put different content into typed messages, and this would not vary regardless of the way in which the messages are delivered. Finally, any such gender effects add noise to our data and thus work against our ability to find evidence for cues. This enhances our confidence in our results.

Our results might explain some patterns in previously published data. For example, Charness and Dufwenberg (2010) offered new data on their hidden action trust game (Charness and Dufwenberg 2006) and found that, in contrast with their original data, the prefabricated statements “I will not roll” or “I will roll” do not promote trust or cooperation. Charness and Dufwenberg indicate that this might be due to the impersonal nature of the message. Another factor might be that these statements are quite short and the perceived effort from the sender is low. The results of our paper suggest that both of these features would make any message, personal or otherwise, less likely to be considered a promise.

Another important example relates to the receivers of promises that include mentions of money. For example, billboards advertising large monetary benefits (discounts or savings) to people who choose to shop at a particular retail location should be aware that such promises may be likely to be broken, and that the reality of the savings may be less than the advertised amount.^32^ Our results indicate that consumers of advertisements should be especially cautious of promises that include specific monetary commitments.

Our study is only one step towards an understanding of this important topic, and is limited in a number of ways. One limitation is that the promises in our environment all relate to money, while in many natural contexts it would be unnatural to refer to money as part of the promise process (e.g., many promises do not involve money). Similarly, we studied a particular game, and different games may lead people to use or to recognize different cues than we discovered, or to use or recognize the same cues differently. Finally, our results were derived from a particular cultural environment. The same games played with different cultural groups may generate different types of cues (e.g., some cultures may be reluctant to use “we” or “us” with strangers.) Indeed, cross-cultural research on deception detection would undoubtedly be very enlightening.

## Electronic supplementary material

Below is the link to the electronic supplementary material.

Supplementary material 1 (PDF 70 kb)

## Footnotes

1 Deception detection is widely studied in Psychology, as discussed in Sect. 2.

2 We are interested in understanding cues used in informal written communication of the sort that people might send in instant messages or other forms of casual (and often electronic) communication. Our focus is not, for example, formal legal documents, which are typically constructed with the goal of reducing ambiguity (at least for those individuals trained in reading the contracts).

3 Through for instance, match.com and many other websites.

4 For anecdotal evidence see “A Million First Dates”, http://www.theatlantic.com/magazine/archive/2013/01/a-million-first-dates/309195/?single_page=true.

5 Communication that has no direct effect on players’ payoffs and is costless and unverifiable.

6 The goal has been to explain why senders are likely to honor their messages even when they incur costs by doing so.

7 The common setups in psychology studies include actors (usually students) who are instructed to tell the truth or a lie, and observers who evaluate the truth of the actors’ statements upon watching videotaped recordings (see Ekman 2009a, b for a short review). For most of those studies, neither the actors nor the observers are incentivized to perform (Zuckerman et al. 1981; Vrij et al. 2004).

8 The subjects were not able to recognize or use all the objective features of the game show, e.g., the relative contribution to the prize.

9 Belot et al. (2012) categorized communication into three different groups: no promise-where no promises are made; voluntary promise-where players voluntarily make promises; and elicited promise-where the subjects were prompted by the game show host to indicate their intention to either cooperate or defect.

10 As noted in Ekman et al. (1999), successful subjects were able to use facial clues to detect liars, as opposed to others who were not able to do so when presented with the same video recordings.

11 Schniter et al. (2012) looked at computer mediated communications and found that apologetic and upgraded messages are more likely to win back trust from the betrayed partners, although those message senders who have previously broken their promises are no more likely to keep their second promises.

12 We denote it Mistress Game because the payoff structure broadly resembles the tradeoffs in a wife (Role A), Husband (Role B), Mistress (Role C) situation. The analogy used here can facilitate understanding of the tradeoffs that each player faces in the game.

13 This game is a modification of an extended three-person trust game with different multipliers for different trustees. Related games include Dufwenberg and Gneezy (2000)—two-person lost wallet game; Charness and Dufwenberg (2006)—two-person trust game with a hidden action; Sheremeta and Zhang (2014) and Rietz et al. (2013)—sequential three person trust game; and Cassar and Rigdon (2011)—three person trust game with one trustee two trustor or one trustor two trustee, and Bigoni et al. (2012)—two person trust game with an add-on dominant solvable game between the trustee and a third player.

14 For in depth analysis of behaviors for all the players in the game, please see Chen and Houser (2014). We would like to highlight that the 2014 manuscript focuses on message senders, while the current paper focuses on the readers of the messages.

15 We left out the B-A messages from the analyses for two reasons: (1) compared with C, B has less incentive to deceive in the game; (2) the actual decisions made by B may be confounded, in that those decisions may not reflect the intent of the messages but rather the messages they later received from C.

16 The authors also implemented other versions of the communication treatment (e.g., only B sends messages to A). These data are reported in Chen and Houser (2014). Here we only focus on the C to B message treatments.

17 It is well understood amongst subjects that they cannot write anything that is self-identifiable, and the experimenter monitors the messages to make sure this rule is followed.

18 The game sessions were also conducted in George Mason University.

19 We collected the first set of messages and evaluations (20 messages from Single and 32 messges from Double, 45 evaluators) in 2013 and second addition set of messages and evaluations (28 messages from Double, 48 evaluators) in 2015.

20 A similar definition was used in Houser and Xiao (2010).

21 In an XH classification game, a group of evaluators is given a list of N messages and a set of categories. Their job is to assign each message to a single category. They are paid for n (n < N) randomly chosen messages whose classifications match a most popular classification respectively.

22 We analyze external observers who did not participate in the game. If they are able to detect deception, this provides evidence that the game’s players may have also been able to do so. Our data do not reveal whether the players in the game were able to detect deception, or the beliefs they held regarding the possibility that they would be deceived.

23 A message is coded as a promise if a majority of the evaluators (more than 50 %) coded the message as such.

24 Our findings regarding promise frequency are consistent with previously reported data. For example, Charness and Dufwenberg (2006) classified 57 % of their messages from B in the (5, 5) treatment as promises; Vanberg (2008) classified 85 % of the messages as promises in No Switch and 77 % of the messages as promises in Switch. Using the same procedure as we do, Houser and Xiao (2010) found that 74 % of the B messages from Charness and Dufwenberg (2006) (5,5) experiment were categorized as promises by the evaluators in their weak promise treatment.

25 We also conducted the Kappa test using all the messages, K = .34, Z = 67.77 and P = .00. The results indicate that we have “fair” (Landis and Koch 1977) amount of agreement amongst evaluators and the level of agreement is significantly higher than chance.

26 To assess whether pooling was appropriate, we performed a Chow test within a Tobit regression analysis. The results indicate that it is appropriate to pool the data from these environments.

27 We also performed a panel data analysis with random individual effects; the results are qualitatively identical. Details are available from the authors on request.

28 For each of the specifications in Table 4, we performed the Chow test. The results suggest that it is appropriate to pool the data, with p-values .15, .21, .38 and .51 respectively.

29 We also performed a panel data analysis with random individual effects, and the results are qualitatively identical. Details available from the authors on request.

30 In Tables 6, 7 and 8, we only include messages classified as promises by majority of the evaluators (>50 %). As a robustness check, we also conducted the same regressions using messages classified as promises by a super majority (>60 %). In doing so, despite losing five observations, the regression results remain almost unchanged. If we include only messages classified as promises by at least two thirds of the evaluators, then we lose 12 observations. The corresponding results remain qualitatively similar to those reported above, but with reduced statistical significance.

31 These results are consistent with the earlier findings reported by Belot et al. (2012).

32 For example, one highway billboard near us reads: “$700 Cash today, the Ca$h Store”. Preceding the “$700” there is an almost entirely unnoticeable “Up to.”
