# Supplementary material for: Promises and lies: can observers detect deception in written messages
Source: Exp Econ. 2016 Jul 8;20(2):396–419. doi: 10.1007/s10683-016-9488-x (PMC5425497; doi:10.1007/s10683-016-9488-x)
Supplement: Supplementary file 1 — Supplementary material 1 (PDF 70 kb) [file 10683_2016_9488_MOESM1_ESM.pdf]

Appendix1-instructions

Thank you for coming! You've earned $5 for showing up on time, and the following instructions will explain your task in this session.

<u>Your task:</u>

You will be given a list of messages. You have two tasks for today's experiment.

<u>Task I</u>

You will evaluate whether each of the messages is:
a. A statement of intent or promise OR
b. Empty Talk

The messages were written by participants in a previous experiment (Experiment I). To evaluate the messages, you need to first understand Experiment I. Instructions for Experiment I begin on page 2. Please read those pages carefully. <u>The message writer is in the role of **subject C.**</u>

It is important for you to know more about how to code the messages before you read the instructions. Here are your specific instructions for how you code the messages.

1) You should code a message as "A statement of intent or promise" if you think at least one of the following conditions is **probably** satisfied.
   a) The writer, subject C, indicates in the message he/she would do something favorable to subject B or refrain from doing something that harms subject B.
   b) The message gives subject B reasons to believe or expect that subject C would do something favorable to subject B or refrain from doing something that harms subject B.
2) You should code a message as "Empty Talk", if the message **probably** does not satisfy any of the above conditions.
3) You should **independently** code all messages. Do not discuss with anyone else in this room about how to code the messages.

<u>Task II</u>

After you have coded all of the messages, you next indicate which decision you believe was made by subject C for each of the messages (either "Left" or "Right").

<u>Your payoff:</u>

For Task I, at the end of the experiment, two messages will be randomly chosen. If your evaluation matches *<u>the most common evaluation</u>* for that message, then you will be paid $5 for that message. Thus, you can earn up to $10 in this part of the experiment.

For Task II, at the end of the experiment, two messages will be randomly chosen. If your guess matches *<u>the actual decision made by the person who wrote that message</u>*, you will be paid $5 for that message. Thus, you can earn up to $10 in this part of the experiment.

Page 2

[Instructions For Experiment I – Part I]

Thank you for participating in today's experiment. The purpose of this experiment is to study how people make decisions in a particular situation. Feel free to ask us questions as they arise, by raising your hand. Please do not speak to other participants during the experiment. You have already received $5 for attending today's experiment. You may also receive additional money, depending on the decisions made (as described below). Upon completion of today's experiment, this additional amount will be paid to you individually and privately.

During the session, you will play versions of the game described below three times. Each time, you will be paired with two other people with whom you have not previously been paired. No participant will ever know the identity of the person with whom he or she is paired. Your earnings in today's experiment will be based on one of the three games. We will determine which of the three games determines your earnings randomly at the end of today's experiment.

Decision Tasks in the Game

In each pair, one person will have the role of A, one will have the role of B, and the other will have the role of C. The amount of money you earn depends on the decisions made in your group.

On the designated decision sheet, each person A will indicate whether he or she wishes to choose IN or OUT. If A chooses OUT, then A and B each receive $5 and C receives $10. We will collect these sheets after A's choices have been indicated. Next, each person B will indicate whether he or she wishes to choose IN or OUT. Note that B will not know whether A has chosen IN or OUT; however, since B's decision will only make a difference when A has chosen IN, we ask B's to presume (for the purpose of making this decision) that A has chosen IN.

If A has chosen IN and B chooses OUT, then B receives $10, and A and C also receive $10. We will collect these sheets from B after the choices have been indicated. Next, each person C will indicate whether he or she wishes to choose LEFT or RIGHT. Note that C will not know whether B has chosen IN or OUT; however, since C's decision will only make a difference when both A and B have chosen IN, we ask C's to presume (for the purpose of making this decision) that both A and B have chosen IN.

If both A and B have chosen IN and C chooses LEFT, then C receives $25, A receives $10 and B receives $20. If C chooses RIGHT, C receives $40, and both A and B receive $0. (All of these amounts are in addition to the $5 show-up bonus.)

This information is summarized in the chart below:

	A Receives	B Receives	C Receives
A chooses OUT	$5	$5	$10
A chooses IN, B chooses OUT	$10	$10	$10
A chooses IN, B chooses IN, C chooses LEFT	$10	$20	$25
A chooses IN, B chooses IN, C chooses RIGHT	$0	$0	$40

Page 3

[Instructions for Experiment I – Part II]

Prior to the decisions by A, B and C, B has an option to send a message to A. Each B receives a blank sheet, on which a message can be written, if desired. These messages will be collected and passed on to their paired As. We will allow time as needed for people to write messages. Please print clearly if you wish to send a message.

In these messages, no one is allowed to identify himself or herself by name or number or gender or appearance. (The experimenter will monitor the messages. Violations (experimenter's discretion) will result in the violator receiving only the $5 show-up bonus, and his or her two partners receiving the average amount received by others who are in the same role). Other than these restrictions, B and C may say anything that they wish in these messages. If you wish to not send a message, simply circle the letter B or C at the top of the sheet.

[Verbal Announcement by the Experimenter After Bs' messages have been passed on to paired Cs]

Now that A has received the message from B, C has an option to send a message to B. Each C receives a blank sheet, on which a message can be written, if desired. These messages will be collected and passed on to their paired Bs. We will allow time as needed for people to write messages. Please print clearly if you wish to send a message.

Page 4

[This is the message sheet that C received to write messages on]

C

You may print a message to B below if you wish.

Appendix 2-decision sheet

Task 1

G Number_________________ Name _________________

The messages that you are to evaluate are on the computer screen. Please write down the message number located at the top left corner of the page for each of the message. **Note: not all the messages are in order and please make sure that the message number and the message you are evaluating are matched.**

Please put an **X** in the ( ), which corresponds with your evaluation of whether the message shows intent or a promise or shows empty talk. Please put only one **X** on option per message.

Msg No. ( ) Intent or a Promise ( ) Empty Talk

Msg No. ( ) Intent or a Promise ( ) Empty Talk

Msg No. ( ) Intent or a Promise ( ) Empty Talk

Msg No.	( ) Intent or a Promise	( ) Empty Talk
Msg No.	( ) Intent or a Promise	( ) Empty Talk
Msg No.	( ) Intent or a Promise	( ) Empty Talk
Msg No.	( ) Intent or a Promise	( ) Empty Talk
Msg No.	( ) Intent or a Promise	( ) Empty Talk
Msg No.	( ) Intent or a Promise	( ) Empty Talk
Msg No.	( ) Intent or a Promise	( ) Empty Talk
Msg No.	( ) Intent or a Promise	( ) Empty Talk
Msg No.	( ) Intent or a Promise	( ) Empty Talk
Msg No.	( ) Intent or a Promise	( ) Empty Talk
Msg No.	( ) Intent or a Promise	( ) Empty Talk
Msg No.	( ) Intent or a Promise	( ) Empty Talk
Msg No.	( ) Intent or a Promise	( ) Empty Talk
Msg No.	( ) Intent or a Promise	( ) Empty Talk
Msg No.	( ) Intent or a Promise	( ) Empty Talk
Msg No.	( ) Intent or a Promise	( ) Empty Talk
Msg No.	( ) Intent or a Promise	( ) Empty Talk
Msg No.	( ) Intent or a Promise	( ) Empty Talk
Msg No.	( ) Intent or a Promise	( ) Empty Talk
Msg No.	( ) Intent or a Promise	( ) Empty Talk
Msg No.	( ) Intent or a Promise	( ) Empty Talk
Msg No.	( ) Intent or a Promise	( ) Empty Talk

Msg No.	( ) Intent or a Promise	( ) Empty Talk
Msg No.	( ) Intent or a Promise	( ) Empty Talk
Msg No.	( ) Intent or a Promise	( ) Empty Talk
Msg No.	( ) Intent or a Promise	( ) Empty Talk
Msg No.	( ) Intent or a Promise	( ) Empty Talk
Msg No.	( ) Intent or a Promise	( ) Empty Talk
Msg No.	( ) Intent or a Promise	( ) Empty Talk
Msg No.	( ) Intent or a Promise	( ) Empty Talk
Msg No.	( ) Intent or a Promise	( ) Empty Talk
Msg No.	( ) Intent or a Promise	( ) Empty Talk

Task II

G Number__________________ Name __________________

The messages on which you are to make a guess are on the computer screen. Please write down the message number located at the top left corner of the page for each of the message. **Note: not all the messages are in order and please make sure that the message number and the message you are making a guess are matched.**

Please put an **X** in the (   ), which corresponds with your guess of whether the person who wrote the message chose LEFT or RIGHT. Please put only one **X** on option per message.

	Your Guess	
Msg No.	( ) LEFT	( ) RIGHT
Msg No.	( ) LEFT	( ) RIGHT
Msg No.	( ) LEFT	( ) RIGHT
Msg No.	( ) LEFT	( ) RIGHT

Msg No.	( ) LEFT	( ) RIGHT
Msg No.	( ) LEFT	( ) RIGHT
Msg No.	( ) LEFT	( ) RIGHT
Msg No.	( ) LEFT	( ) RIGHT
Msg No.	( ) LEFT	( ) RIGHT
Msg No.	( ) LEFT	( ) RIGHT
Msg No.	( ) LEFT	( ) RIGHT
Msg No.	( ) LEFT	( ) RIGHT
Msg No.	( ) LEFT	( ) RIGHT
Msg No.	( ) LEFT	( ) RIGHT
Msg No.	( ) LEFT	( ) RIGHT
Msg No.	( ) LEFT	( ) RIGHT
Msg No.	( ) LEFT	( ) RIGHT
Msg No.	( ) LEFT	( ) RIGHT
Msg No.	( ) LEFT	( ) RIGHT
Msg No.	( ) LEFT	( ) RIGHT
Msg No.	( ) LEFT	( ) RIGHT
Msg No.	( ) LEFT	( ) RIGHT
Msg No.	( ) LEFT	( ) RIGHT
Msg No.	( ) LEFT	( ) RIGHT
Msg No.	( ) LEFT	( ) RIGHT
Msg No.	( ) LEFT	( ) RIGHT

Msg No.	( ) LEFT	( ) RIGHT
Msg No.	( ) LEFT	( ) RIGHT
Msg No.	( ) LEFT	( ) RIGHT
Msg No.	( ) LEFT	( ) RIGHT
Msg No.	( ) LEFT	( ) RIGHT
Msg No.	( ) LEFT	( ) RIGHT
Msg No.	( ) LEFT	( ) RIGHT
Msg No.	( ) LEFT	( ) RIGHT
Msg No.	( ) LEFT	( ) RIGHT

Quiz for the experiment I instruction:

1. If you are in the role B, before you make your decision, will you know the decision of A?

2. If you are in the role C, will you know the decision of B before you make your decision?

3. If you are in the role A, and you choose OUT, what is your payoff for this game?

4. If you are in the role B and you choose IN, assume that the A, C that you are paired with choose, IN and LEFT, what is your payoff for the game? What is A’s payoff? What about C’s?

5. Did Cs know at the beginning of the game that they were able to send a message to B?

Quiz for the tasks today:

6. What is the maximum you can earn for today’s tasks? (Excluding the show-up bonus)
